# Novel Biomarkers of Physical Activity Maintenance in Midlife Women: Preliminary Investigation

**DOI:** 10.1089/biores.2018.0010

**Published:** 2018-04-01

**Authors:** Kelly A. Bosak, Vlad B. Papa, Morgan G. Brucks, Cary R. Savage, Joseph E. Donnelly, Laura E. Martin

**Affiliations:** ^1^ School of Nursing, University of Kansas Medical Center, Kansas City, Kansas.; ^2^ Hoglund Brain Imaging Center G001, University of Kansas Medical Center, Kansas City, Kansas.; ^3^ Center for Brain, Biology and Behavior, University of Nebraska, Lincoln, Nebraska.; ^4^ Cardiovascular Research Institute, University of Kansas Medical Center, Kansas City, Kansas.; ^5^ Structure and Function Unit, Hoglund Brain Imaging Center, University of Kansas Medical Center, Kansas City, Kansas.

**Keywords:** biomarkers, brain–behavior connection, midlife women, neuroscience, physical activity maintenance

## Abstract

The precision health initiative is leading the discovery of novel biomarkers as important indicators of biological processes or responses to behavior, such as physical activity. Neural biomarkers identified by magnetic resonance imaging (MRI) hold promise to inform future research, and ultimately, for transfer to the clinical setting to optimize health outcomes. This study investigated resting-state and functional brain biomarkers between midlife women who were maintaining physical activity in accordance with the current national guidelines and previously acquired age-matched sedentary controls. Approval was obtained from the Human Subjects Committee. Participants included nondiabetic, healthy weight to overweight (body mass index 19–29.9 kg/m^2^) women (*n* = 12) aged 40–64 years. Control group data were used from participants enrolled in our previous functional MRI study and baseline resting-state MRI data from a subset of sedentary (<500 kcal of physical activity per week) midlife women who were enrolled in a 9-month exercise intervention conducted in our imaging center. Differential activation of the inferior frontal gyrus (IFG) and greater connectivity with the dorsolateral prefrontal cortex (dlPFC) was identified between physically active women and sedentary controls. After correcting for multiple comparisons, these differences in biomarkers of physical activity maintenance did not reach statistical significance. Preliminary evidence in this small sample suggests that neural biomarkers of physical activity maintenance involve activations in the brain region associated with areas involved in implementing goal-directed behavior. Specifically, activation of the IFG and connectivity with the dlPFC is identified as a neural biomarker to explain and predict long-term physical activity maintenance for healthy aging. Future studies should evaluate these biomarker links with relevant clinical correlations.

## Introduction

The Precision Health Initiative emphasizing individualized healthcare is leading the discovery of novel biomarkers. Objectively measured biologic, physiologic, and “omic” (e.g., genomic, metabolomic, proteomic) biomarkers are the underpinnings of health conditions and their treatments.^1^ Noninvasive neural biomarkers identified by magnetic resonance imaging (MRI) are gaining recognition as important indicators of biological processes or responses to behavior,^2^ such as physical activity. These biomarkers provide information for identifying individuals at risk for disease and allow resources to be focused on those who will benefit most from intensive intervention or treatment. As proposed in this preliminary investigation, neural biomarkers of physical activity maintenance and inactivity in midlife women hold promise to inform future research, and ultimately, for transfer to the clinical setting to optimize healthy aging.

### Neural biomarkers of physical activity maintenance

Some women remain engaged in regular physical activity throughout midlife and beyond. Maintenance of physical activity is affected by biological predisposition and pervasive cues in the environment.^3^ Physical activity has been extensively studied and is accepted as a major determinant of health.^4^ Physical activity confers statistically significant and clinically meaningful reductions in the burden of cardiovascular disease, which remains the leading cause of death for both men and women nation-wide, and reduces the risk of diabetes, hypertension, and some forms of cancer.^5–7^ Physical activity also enhances mental health,^4,8–10^ and improves muscle, bone, and joint function necessary for aging adults to remain independent in their home and community in later years. Furthermore, the impact of inactivity and patterns of sedentary time accumulation on health is recognized as distinct from physical activity, increasing risk for chronic disease and negatively impacting quality of life.

Despite the importance of regular physical activity, it is difficult for many individuals to maintain or increase physical activity in midlife and beyond. Women who enter midlife with a higher level of physical activity and maintain it or those who increase their physical activity after menopause have a reduced tendency to experience adverse outcomes than their less-active peers.^11^ Incidentally, research shows that for many women, a reduction in spontaneous physical activity parallels the decline in estrogen.^12,13^ This can lead to a sedentary lifestyle that women may not be mindfully aware is occurring. The effects of estrogen depletion on brain function may be one of the mechanisms contributing to sedentary behavior.

Neuroimaging allowed us to characterize the biomarkers of resting-state connectivity and functional activation (i.e., percent signal change from baseline) in the brain during a cognitive task associated with maintenance of physical activity in midlife women. The novel biomarkers identified in this investigation with midlife women may explain and predict cognitive control and self-regulation associated with physical activity maintenance.

### Resting-state MRI

Resting-state cognitive function has generated considerable attention due to its clinical relevance.^14^ The resting state refers to functional connectivity of the brain at rest, also referred to as the baseline or “default mode” of brain function that is suspended during goal-directed activities.^15^ In a previous study comparing weight reduction interventions (dieting or gastric banding) differences were found in biomarkers of connectivity in the resting-state MRI (rsMRI) between satiety and weight reduction interventions in the temporal gyrus and insula.^16^ Resting-state connectivity in these regions is associated with emotion processing and salience. Following a meal, these regions were activated differentially between intervention groups. Activation of these regions of interest in the current study is identified as a biomarker of cognitive control and self-regulation promoting physical activity maintenance.

### Goal-directed functional activation

Conventional research suggests that the use of cognitive control or self-regulation strategies enhance an individual's belief in their personal capabilities for physical activity, which in turn, results in improved physical activity maintenance.^17^ A study using neuroimaging with older adults (mean age 66.4 years) randomly assigned to either a walking group or a flexibility, toning, and balance group identified biomarkers of improved adherence to physical activity. Activation of the prefrontal cortex (PFC) was associated with higher baseline executive control, and thus, the ability to persist with physical activity.^18^

de Wit et al. also identified neural biomarkers, including activation of the ventromedial prefrontal cortex (vmPFC), during the learning phase of a goal-directed cognitive task.^19^ In our previous study of neural biomarkers, activation during the goal-directed learning phase showed a positive correlation with body mass index (BMI) in the dorsal medial prefrontal cortex (dmPFC) and a negative correlation with BMI in the insula/inferior frontal gyrus (IFG).^20^ During the implementation of goal-directed behavior, brain biomarkers in the dorsolateral prefrontal cortex (dlPFC) negatively correlated with BMI.^20^ These findings indicate that biomarkers in the regions associated with cognitive control differed between overweight and healthy weight women during goal-directed learning. Biomarkers of emotion processing, planning, and self-regulation were identified in healthy weight in the anterior insula/IFG regions to a greater degree than overweight women during learning and implementation of goal-directed behavior. Further, a biomarker was identified in the cognitive control region with overweight women while learning; however, this biomarker was not detected during implementation, which indicates greater difficulty transforming goals into action (e.g., maintain physical activity).

In weight reduction studies, brain biomarkers were identified in the vmPFC and dlPFC, while participants made decisions about food correlated with weight loss.^21,22^ Furthermore, a preliminary study focusing on food motivation identified differential functional MRI (fMRI) biomarkers in the vmPFC in healthy weight, compared with obese participants.^23^ Overall, these findings demonstrate that the PFC is a region involved in cognitive control and self-regulation and processing goal-directed stimuli related to health behaviors. It is posited that activations or biomarkers in these areas of the PFC are associated with goal-directed behavior, and will show strong connectivity in the context of maintenance of physical activity.

### Study aims

The aims of this preliminary study were to investigate: (1) brain biomarkers in the resting state of the left dlPFC in midlife women maintaining physical activity, compared to a previously acquired age-matched inactive control group.^24^ We hypothesized that in the resting state, whole brain differences would be identified in physically active midlife women, compared to inactive controls; and (2) functional brain biomarkers during goal-directed task learning and implementation phases between the physical activity maintenance group, and a previously acquired age-matched inactive control group.^20^ Based on our previous research, we hypothesized that biomarkers during the functional task would be identified in regions of the PFC (dmPFC, dlPFC, and insula) associated with self-regulation and goal-directed behavior in physically active women, compared with inactive controls.

## Materials and Methods

Approval for this study was obtained from the Human Subjects Committee at the University where this study was conducted. Participants included nondiabetic, healthy weight to overweight (BMI 19–29.9 kg/m^2^) women (*n* = 12), aged 40–64 years. In addition, participants recruited were required to be exercising at moderate (or greater) intensity for 30 min or more on most days of the week for at least the past 6 months, based on the current national physical activity guidelines.^25,26^ We used participant's data from those enrolled in our previous fMRI study^20^ and baseline rsMRI data from a subset of inactive (<500 kcal of physical activity per week) women aged 40–55 who were enrolled in a 9-month exercise intervention^24^ conducted in our imaging center.

We sent out broadcast email messages to university faculty and staff on campus. Based on our previous successful recruitment of adult participants using this method, we were able to accrue the targeted number for this pilot study over ∼3 months. All participants underwent rsMRI and fMRI scan at the one-time visit. Two participants were excluded from fMRI data analysis due to low accuracy (<50% correct) on the cognitive task conducted in the scanner. Participants were required to be able to read and speak English, as the cognitive task was only available in English. Potential participants were excluded if they reported any contraindications to MRI scanning (e.g., permanently implanted metals not approved for 3 T MRI, history of myocardial revascularization, percutaneous coronary intervention, coronary artery bypass grafting, or other major surgery, indicating the potential for implanted metal). Participants were excluded if they were taking psychotropic medication (e.g., antidepressants, stimulants) or thyroid medication due to potential effects on brain activations. Participants taking beta blockers were also excluded due to potential for alterations in cerebral blood flow.

This study was limited to females due to the differences between the sexes in metabolic regulation and associated conditions of midlife. Cardiovascular disease morbidity and mortality continues to increase in females, with a marked increase in midlife following menopause.^27,28^ Although not assessed in this study, a decline in spontaneous physical activity was found to parallel the estrogen depletion in midlife women with little conscious awareness that this is occurring.^13^ This indicates neurohormonal changes and the need for neuro biomarker identification to overcome these adverse effects and to promote positive health behaviors.

Scanning was conducted at the free-standing easily accessible research-dedicated facility on our university campus. The visit included a resting-state scan (with eyes open), and a functional scan using the goal-directed cognitive task in the scanner. Two potential participants did not qualify for the study, due to previous surgery and the potential for implanted metal.

The cognitive task used in the scanner was based on the work of de Wit et al.^19^ and used in the principal investigators' previous study (for a full description of this task see Bosak and Martin^20^). This procedure tests goal-directed cognitive processes during a learning phase and an implementation phase. Brain responses were investigated in the first phase, while participants learned a goal-directed task, and in the second phase, while they implemented what they learned. As described in our previous study, the task was modified slightly for the midlife adult age group to allow additional learning time by increasing the number of learning sequences. Participants received a demonstration of the task before going into the scanner and were instructed to respond to an object (commonly recognized fruits) on a screen by pressing a left or right response key associated with that object. Correct responses led to the outcome-associated object and points were added to the participant's overall score. Participants were not told which key was the correct response, rather they learned as the task proceeded. The quicker a correct response was made, the more points were received. Participants were instructed to earn as many points as possible during the task. A questionnaire was given at the end of the scan to test if the correct associations were learned. Participants completed self-report surveys and the fMRI scan at the Hoglund Brain Imaging Center in a session lasting ∼2 h. This general task of goal-directed behavior can be translated to a variety of contexts and health conditions to investigate health behavior.

### Demographics

The demographic characteristics of the midlife women who maintain physical activity and the inactive control group demographics acquired from two previous studies are shown in Table 1.

**Table 1.** **Demographic Characteristics by Sample**

	Age	Race	Ethnicity	
Group	*M* (range)	*n* (%)	*n* (%)	BMI, kg/m^2^ (range)
Physical activity maintenance (*N* = 12)	52 (44–64)	Caucasian 12 (100)	Not Hispanic 7 (58.3)	26.5
			Other 4 (33.3)	
			Missing 1 (8.4)	
Sedentary control (rsMRI) (*N* = 12)	46 (42–50)	Caucasian 12 (100)	Not Hispanic 11 (91.6) Other unknown 1 (8.4)	30.22 (23–40.6)
Sedentary control (fMRI) (*N* = 19)	51.3 (47–55)	Caucasian 17 (89.5) African American 2 (10.5)	Not Hispanic 19 (100)	25.7 (20–37.1)

BMI, body mass index; fMRI, functional magnetic resonance imaging; rsMRI, resting-state MRI.

### Data acquisition

Scanning was performed on a 3 T (indicates magnet strength) Siemens Skyra scanner using standard scanning parameters. T1 weighted three-dimensional magnetization prepared rapid acquisition gradient echo images were obtained (repetition time/echo time [TR/TE] = 23/2 msec, flip angle = 9°, field of view = 256 mm, matrix = 256 × 176, slice thickness = 1.2 mm). These images provided slice localization for functional scans, Talairach (brain coordinates map) transformation, and coregistration with fMRI data. Resting-state data were acquired while participants were instructed to keep their eyes open and focused on a fixation mark at the center of the screen. Resting-state scanning parameters included gradient echo blood oxygen level-dependent (BOLD) sequences of 43 contiguous slices at a 40° angle to the anterior commissure/posterior commissure (AC/PC) line (TR/TE = 2500/25 msec, flip angle = 90°, matrix = 80 × 90, slice thickness = 3 mm, in plane resolution = 2.9 mm, for 144 data points). Task-based fMRI data were acquired while participants completed the goal-directed learning and implementation tasks. fMRI scanning parameters included six functional gradient echo BOLD sequences of 50 contiguous slices at a 40° angle to the AC/PC line (TR/TE = 3000/25 msec, flip angle = 90°, matrix = 80 × 80 slice thickness = 3 mm, in plane resolution = 2.9 mm, for 80 data points). All resting-state and task-based fMRI scans were acquired at a 40° angle to the AC–PC line to optimize vmPFC signal by minimizing susceptibility artifact and all participants were positioned in the scanner so that the angle of the AC–PC plane was between 17° and 22°.^20,24^ These are considered to be standard fMRI parameters.

### Task data analysis

Data processing and statistical analyses were performed using Analysis of Functional NeuroImages (AFNI; Medical College of Wisconsin). Preprocessing steps for fMRI included alignment, spatial smoothing and normalization, and motion correction. The fMRI images were aligned to the third slice in each run to correct for motion, and time points during which participants moved more than 0.3 mm within a temporal resolution (TR =3000 msec) were censored. The images were spatially smoothed with a 4-mm full width at half maximum (FWHM) Gaussian blur. Anatomic images were aligned to functional images. Participants' anatomical images were normalized to Talairach stereotaxic space using AFNI's automated algorithm, and this transformation was applied to the participants' functional scans. The primary data analysis focused on whole-brain voxelwise *t*-test to determine brain biomarker differences between women who maintain physical activity and inactive women during goal-directed learning (first three fMRI runs) and implementation of goal-directed behavior (last three fMRI runs) by comparing congruent and control conditions to the incongruent conditions. Previous research using this paradigm identified different areas of the brain involved in resolving tasks associated with congruent, control, and incongruent conditions.^19^

### Resting state data analysis

Data preprocessing (cleaning) and statistical analysis were conducted using AFNI.^29^ Preprocessing analysis scripts were built using afni_restproc.py.^30^ Preprocessing included removing the first four volumes, removing any transient signal, slice time correction, and coregistered all functional data to the first volume. Nuisance variables were measured (i.e., six motion parameters [three translations, three rotations], average ventricle signal, and average local white matter signal [15 mm spherical neighborhood, 3dLocalstat]) and removed from the signal time course using multiple regression. The residual time course images were then smoothed with a 6 mm FWHM Gaussian kernel, resampled to a 2 mm × 2 mm × 2 mm grid, and spatially normalized to Talairach stereotaxic space.^31^ In addition to controlling for six motion parameters, further motion correction procedures (i.e., scrubbing) were used to reduce possibility type I error related to motion.^32,33^ Time points with greater than 0.3 mm motion were censored.

### Seed region selection and identification

**FIG. 1.**
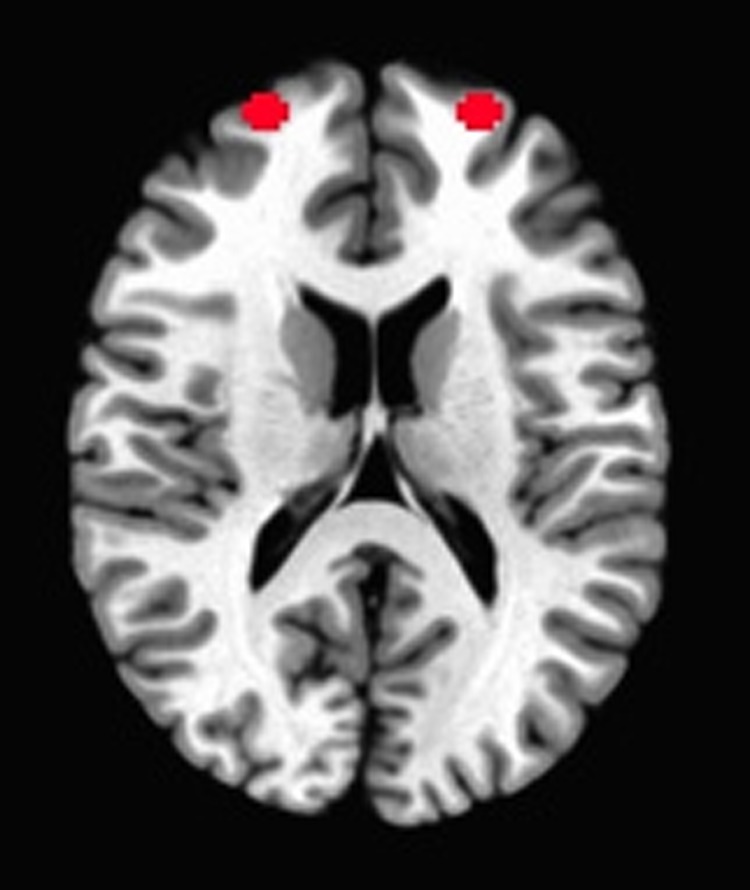
Bilateral dorsolateral prefrontal seed region. R.dlPFC seed (*x*, *y*, *z* = 23, 56, 15), L.dlPFC seed (*x*, *y*, *z* = −23, 56, 15). dlPFC, dorsolateral prefrontal cortex.

Bilateral dorsolateral prefrontal seed region was chosen to examine connectivity between brain regions associated with goal-directed behavior and the rest of the brain. Specifically, a left dlPFC seed region with 5 mm radii (Talairach *x*, *y*, *z* = −23, 56, 15) and the mirrored right dlPFC (*x*, *y*, *z* = 23, 56, 15) were chosen based on analysis of the functional activation data from our previous study (Fig. 1).^20^

### Functional connectivity analyses

At the subject-level, the bilateral seed time series were constructed separately by calculating the average time series over the voxels within each of the seed regions. Multiple regression analysis was used to produce a correlation map for each seed of the correlations (*r*-values) between the seed time series and each voxel in the brain. Correlation values (*r*-values) were then transformed to *z*-scores. An independent samples *t*-test was used to identify voxels exhibiting group differences in spontaneous BOLD fluctuations correlated with each seed region.

### Correction for multiple comparisons

To correct for multiple comparisons, the spatial autocorrelation function option was used in AFNI's 3dFWHMx to estimate intrinsic smoothness and 3dClustSim to estimate probability of false positives. Cluster size corrections for multiple comparisons were achieved with voxel-wise *p* < 0.001 and a minimum cluster size of 235 mm^3^ (15 voxels [2.5 × 2.5 × 2.5]) for the whole-brain task and 560 mm^3^ (70 voxels [2 × 2 × 2]) for rsMRI. In addition, activations within *a priori* regions, which were found in our previous study, are reported with an uncorrected voxel-wise *p* < 0.001.

## Results

### rsMRI results

**FIG. 2.**
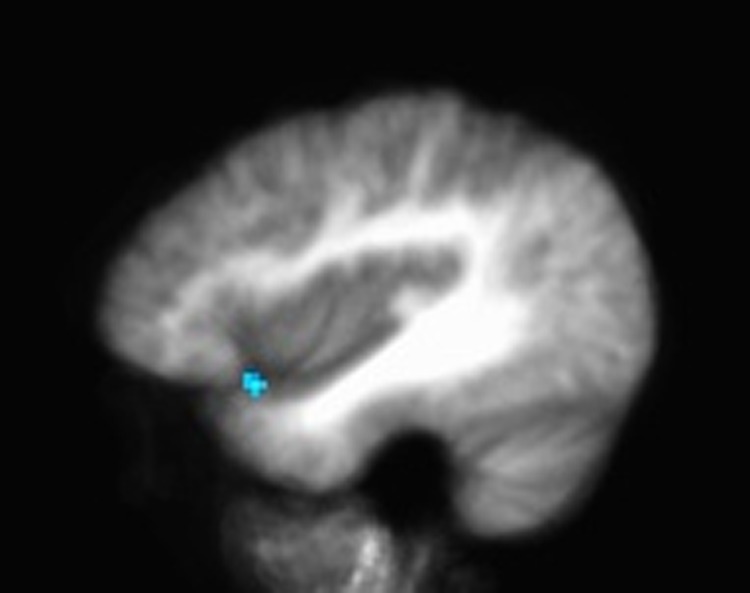
Connectivity between the left dlPFC and inferior frontal gyrus (*x*, *y*, *z* = −39, 15, −12; 104 mm^3^).

No significant whole-brain differences were found between groups comparing connectivity between the right or left dlPFC and the rest of the brain. However, a trend suggested greater connectivity between the left dlPFC and IFG (*x*, *y*, *z* = −39, 15, −12; 104 mm^3^) in the physical activity group compared with the inactive control group (Fig. 2).

### fMRI results

#### Goal-directed learning

**FIG. 3.**
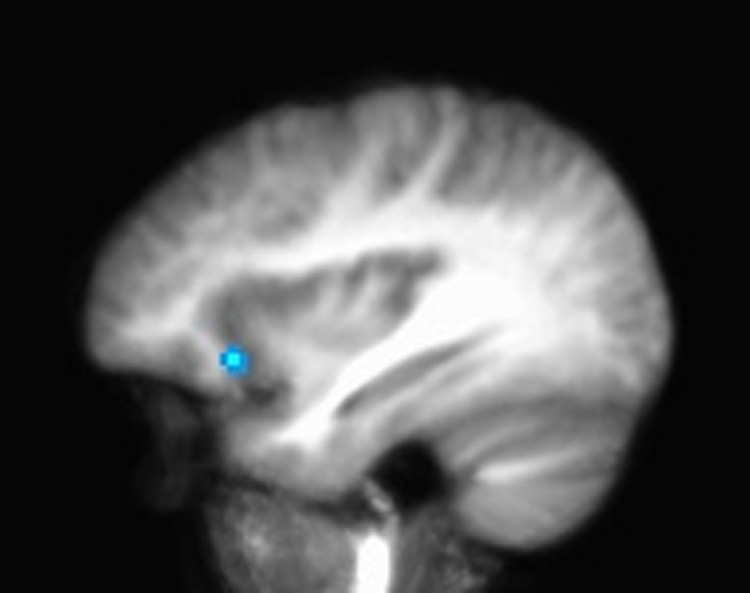
Potential biomarker in right inferior frontal gyrus (*x*, *y*, *z* = 34, 21, −6; 62 mm^3^).

A trend was found in the right IFG (*x*, *y*, *z* = 34, 21, −6; 62 mm^3^) demonstrating a potential biomarker with greater differentiation between conditions for physical activity but not inactive participants. In this small sample, these differences did not reach significance when correcting for multiple comparisons (Fig. 3).

#### Goal implementation

During the implementation phase of the cognitive task, no biomarkers were identified, as no significant differences or trends were found between the physical activity maintenance group and inactive participants.

#### Behavioral results: goal-directed learning phase and implementation phase

No significant differences were found in accuracy on the task between midlife women maintaining physical activity and inactive age-matched controls. In the goal-directed learning phase, no significant differences in accuracy were found between groups (inactive: mean = 80%, standard deviation [SD] = 10%; physical activity: mean = 81%, SD = 12%). In the goal implementation phase, the physical activity group showed a trend toward higher accuracy than the inactive group [*t*(27) = 15.33, *p* = 0.06; inactive: mean = 61%, SD = 8%; physical activity: mean = 66%, SD = 3%].

## Discussion

Physical activity in midlife has considerable benefit for preventing or delaying chronic health conditions and promoting independence in later years. Despite these benefits, it is difficult for many adults to maintain physical activity as recommended and levels decline throughout midlife. Neuroimaging methods allowed investigation of novel neural biomarkers of physical activity and inactive behavior. This cross-sectional study compared resting-state and functional goal-directed brain responses in midlife women who were maintaining physical activity in midlife, compared with previously acquired age-matched inactive controls. This research identified potential biomarkers of physical activity maintenance.

Differences were seen in resting-state connectivity between goal-directed areas of the PFC, specifically the dlPFC, associated with implementing goal-directed behavior^34^ identified in our previous study and the IFG, related to emotion processing and salience.^35^ In our small sample, the trend toward higher accuracy in the physical activity maintenance group neared significance [*t*(27) = 15.33, *p* = 0.06]. This indicates that differential activation of the IFG and greater connectivity with the dlPFC between physically active women and inactive women may be a biomarker of physical activity maintenance.

Participants were recruited by broadcast email and responded to the invitation based on their self-reported adherence to the criteria in accordance with the current national physical activity guidelines for adults,^26^ which we acknowledge can be considered a limitation. We used the data from a group of inactive controls from previous research studies to conserve resources. Another limiting factor of this study was the inability to analyze age as a covariate in the fMRI and rsfMRI analyses due to the small sample size. In addition, we acknowledge that groups were not matched on menopausal status, estradiol level, mental health status, or mood. Variables that impact physical activity and neuroimaging are important to consider for inclusion in a future larger study.

In this cross-sectional investigation with a small sample of midlife women, the results suggest that neural biomarkers of physical activity maintenance involve activations in the brain region associated with areas involved in implementing goal-directed behavior. Specifically, activation of the IFG and connectivity with the dlPFC are identified as neural biomarkers to explain and predict long-term physical activity maintenance. Future studies should evaluate these biomarker links with relevant clinical correlations.

This research provides the basis for further investigation of the neural biomarkers of physical activity maintenance and inactive behavior in midlife women. Neural biomarkers are part of an expanding science that in combination with conventional biomarkers may be useful for translation to the clinic setting to prevent or delay chronic conditions and promote healthy aging. Ultimately, this research will inform future clinical interventions to motivate and support health behavior change and improve outcomes for those individuals who will benefit most from intensive physical activity intervention.

## Abbreviations Used

AC/PC: anterior commissure/posterior commissure
AFNI: Analysis of Functional NeuroImages
BMI: body mass index
BOLD: blood oxygen level dependent
dlPFC: dorsolateral prefrontal cortex
dmPFC: dorsal medial prefrontal cortex
fMRI: functional MRI
FWHM: full width at half maximum
IFG: inferior frontal gyrus
MRI: magnetic resonance imaging
PFC: prefrontal cortex
rsMRI: resting-state MRI
SD: standard deviation
TR/TE: repetition time/echo time
vmPFC: ventromedial prefrontal cortex
